# Citrus Genomics

**DOI:** 10.1155/2008/528361

**Published:** 2008-05-19

**Authors:** Manuel Talon, Fred G. Gmitter Jr.

**Affiliations:** ^1^Centro de Genómica, Instituto Valenciano de Investigaciones Agrarias (IVIA), 46113 Moncada, Valencia, Spain; ^2^Citrus Research and Education Center (CREC), University of Florida, IFAS, Lake Alfred, FL 33850, USA

## Abstract

Citrus is one of the most widespread fruit crops globally, with great economic and health value. It is among the most difficult plants to improve through traditional breeding approaches. Currently, there is risk of devastation by diseases threatening to limit production and future availability to the human population. As technologies rapidly advance in genomic science, they are quickly adapted to address the biological challenges of the citrus plant system and the world's industries. The historical developments of linkage mapping, markers and breeding, EST projects, physical mapping, an international citrus genome sequencing project, and critical functional analysis are described. Despite the challenges of working with citrus, there has been substantial progress. Citrus researchers engaged in international collaborations provide optimism about future productivity and contributions to the benefit of citrus industries worldwide and to the human population who can rely on future widespread availability of this health-promoting and aesthetically pleasing fruit crop.

## 1. INTRODUCTION

Citrus is one of the most important and widely grown of the fruit crops, with total global production reported to be 105.4 million tons in 2004-2005 [1]. Citrus fruit is produced throughout the tropical and subtropical regions of the world, where the winter temperatures are adequate for tree survival and avoidance of freeze devastation, and where there is sufficient water and suitable soils to support tree growth and fruit production. The most significant production areas are found in the Americas (led by Brazil, the United States, Mexico, and Argentina), the Mediterranean basin (led by Spain, Italy, Egypt, and Turkey), and the south and east Asian regions (led by China, India, and Japan). Citrus production, whether for processed or fresh fruit products, from the largest producing countries is an important commodity for global trade and of tremendous economic value and impact. However, there is much citrus production of great importance to local national and regional economies and of value to the nutritional needs of people in less developed nations; this is born out, for example, by the fact that sweet oranges (*Citrus sinensis* L. Osb.) are reported to be grown in 114 countries, grapefruit (*Citrus paradisi* Macf.) and pummelos (*Citrus maxima* Merr.) in 74 countries, and lemons/limes (*Citrus limon* [L.] Burm. F./*Citrus aurantifolia* [Christm] Swing.) in 94 different countries [2].

In addition to the use as a food or beverage source, citrus products from some of the wild species not grown commercially are also of value as agents of traditional medicinal and sanitary utilization [3]. Several closely related genera have varying degrees of sexual compatibility with *Citrus*, some of which produce edible fruit for commerce (e.g., the kumquats, *Fortunella* [Swing.]) and others that possess traits of economic value for rootstock and scion improvement (e.g., the trifoliate orange or *Poncirus trifoliata* [L.] Raf.). Despite the diversity of fruit types, however, nearly 70% of the world's citrus production is sweet orange.

Given the tremendous extent and value of citrus production, it may be somewhat surprising on first consideration that nearly all of the major scion and rootstock cultivars utilized in much of the world have not arisen as a consequence of systematic and targeted breeding programs. Rather, they have arisen spontaneously as seedling and/or bud sport mutations or by introduction and trials of materials from one location to another [4–6]. The reasons for the low level impact of traditional breeding approaches to genetic improvement of this major fruit crop are related to the peculiarities of citrus reproductive biology and the fairly unique aspects of the taxonomic relationships of the major cultivar groups [7]. Citrus seedlings are subject to juvenile periods ranging from one to as many as 20 years, though typically they will flower and fruit within 3–7 years, depending on species. Even after first flowering, it is common for fruit traits to be atypical of later characteristics as scion lines mature. One consequence of juvenility is the obvious delay between hybridization and selection for desired characteristics; however, a secondary consequence is the requirement of large unit areas of land to grow substantially large individual hybrids, thereby increasing the cost of maintenance in the field and limiting the number of families and individuals within families that can be grown. Further, many of the commercial citrus types produce polyembryonic seeds through nucellar embryony, yielding seedlings that are essentially clones of the maternal parent. These embryos arise autonomously prior to anthesis and their development to maturity follows normal pollination and endosperm development [8]. These nucellar embryos most frequently grow much more vigorously than any zygotic embryos and, consequently, the frequency of true zygotics is extremely low. Finally, it is important to recognize that several of the so-called “species” of economic significance (e.g., *C. sinensis*, *C. paradisi*, and *C. limon*) are not biologically defined species; the cultivars in these groups represent accumulated somatic mutations identified over centuries through on-tree or nucellar seedling mutations [9]. Further, some cultivar groups within other species, such as the Clementine and Satsuma mandarins, are likewise the result of somatic mutations and not a consequence of hybridization. Market and consumer expectations and demands for specific commodities (e.g., sweet oranges, grapefruit, lemons, Clementines, and Satsumas) thereby limit the possibilities for genetic improvement within these cultivar groups because the commodities must meet the consumers' expectations and concepts related to fruit traits. These needs and the narrow germplasm bases actually represented within these cultivar groups, along with the reproductive factors, have precluded breeding as a strategy for cultivar development and improvement. The exceptions to this are pummelos (*C. maxima*), the development of new types of mandarin hybrids (using selections that produce monoembryonic seeds containing true zygotic embryos), and rootstock breeding, where hybridization and selection are viable and productive approaches. In these cases, however, limited genetic understanding of the inheritance and control of critical traits remains a substantial issue.

With globalization of citrus production and increased human travel throughout the world, particularly devastating citrus diseases have been rapidly spreading, thus threatening the viability and the very future of citrus production globally. This is the case with much of agriculture; increasing human populations and urban development have forced citrus production to less desirable regions where environmental factors present greater challenges to sustained production. Increased demands for water resources that follow increased populations and urbanization likewise limit the availability of water resources with adequate quality to maintain tree growth and production. There exist genetic resources to address most of these challenges. However, the genetic challenges and the lack of understanding of the fundamental mechanisms underlying these critical traits, as described above, present tremendous impediments to the progress needed to incorporate needed genes and alleles and to devise the appropriate strategies for the continued production and economically feasible availability of citrus fruits to the future world population. In this context, the advent of genomic science and the powerful new tools that are being developed and utilized for citrus improvement take on critical significance. This article will review the progress that has taken place thus far in the development and application of genomic information for citrus improvement and present the current status of research and future directions envisioned.

## 2. LINKAGE MAPPING

*Citrus* and the closely related genera are partially sexually compatible in varying degrees; they are primarily diploid with a few known triploids and occasional tetraploid forms (2*n* = 2*x* = 18), and they possess fairly small genomes (e.g., sweet orange has been said to be around 367 Mb, or approximately three times that of *Arabidopsis* [10]). As such, the citrus species should be amenable to many of the commonly used techniques and approaches related to genomic research, including genetic and physical mapping, full genome sequencing, and functional genomics studies aimed at unraveling the complexities of key traits of interest. Because of the valuable characteristics within some of the related genera that are absent from *Citrus*, particularly cold tolerance and resistance to citrus canker (caused by *Xanthomonas axonopodis* pv. *citri*) from kumquat (*Fortunella*) and multiple stress-tolerant and disease-resistance traits from *P. trifoliata* (including freeze avoidance, and resistance to citrus tristeza virus (CTV), *Phytophthora*, and citrus nematode [*Tylenchulus semipenetrans*]), many of the genetic mapping projects and some of the physical mapping as well have focused on *P. trifoliata* through intergeneric hybrids with *Citrus*.

Citrus breeders and geneticists have long desired to have linkage maps that empower selection schemes based on easily scored, neutral molecular markers rather than relying on frequently difficult, time-consuming, and inefficient approaches based on phenotypic characterizations. Indeed, genetic linkage maps have been produced across the past two decades with increasing value and resolution, as the evolution of new marker systems has taken place. The first published report of linkage mapping in citrus (using a small intragenic family of *Citrus*, and a larger *Citrus* × *Poncirus* family) was based on leaf isozymes [11]; five markers were found that defined two linkage groups, and significantly this was also the first report of linkage distortion which has been a common feature of the many intergeneric mapping efforts that followed subsequently. As RFLP technology became commonly applied in genetic studies, citrus scientists began to incorporate it together with isozyme methods, and new maps resulted first with 35 markers in 8 linkage groups covering 314 cM within a citrus backcross family [12], followed by maps with 52 and 35 markers each, defining 11 and 10 linkage groups and 533 and 351 cM, in an intergeneric backcross family and a population derived from crossing two individual intergeneric F1 hybrids, respectively [13, 14]. As new marker systems were developed, the maps produced from each of these families were further populated first by RAPDs [15], thereby increasing the number of markers from Durham's map from 52 to 189, decreasing the number of linkage groups to 9, and more than doubling genomic coverage to 1192 cM. Sankar and Moore [16] increased marker coverage in the same map to 310 markers through use of ISSRs. In similar fashion, Jarrell's map was improved through incorporation of SSR loci [17], and then further by ISSR marker development to 156 markers defining 16 linkage groups across 701 cM [18]. AFLP markers were first reported to be used for citrus genetic mapping in 1998 by de Simone et al. [19] and in 1999 by Ling et al. [20] (also elaborating the Durham map). Many other whole genome maps have been produced as well as trait specific maps identifying single gene and QTL regions of significance; these have been summarized by Chen et al. [21]. It is through the latter category of trait specific mapping that some of the promise of genomic science for citrus genetic improvement is being pursued and realized, including selection of disease resistant and environmental stress tolerant hybrids in rootstock breeding programs, and targeted gene cloning projects aimed at providing potential solutions to serious disease problems.

Although higher throughput and increased marker density became possible through application of RAPD, AFLP, and ISSR techniques, these systems were of limited value in comparative genomic studies and in utilization for marker-assisted selection (MAS) methods because of the dominant nature of the markers and their low portability among populations. RFLPs, SCARs, CAPS, SSRs, and SNPs are obviously much more desirable for broad applications, and the citrus research community has been developing these resources over time. SCAR markers for citrus were developed first by Deng et al. [22], based on RAPD markers that were closely linked to *Ctv*, a gene for CTV resistance from *P. trifoliata* [23]. Polymorphism could not be revealed by some of these SCAR markers without restriction of amplified products, thus they were converted to CAPS. In 1999, García et al. [24] likewise used CAPS together with RFLPs, RAPDs, and isozymes to map genes in *Citrus* and *Poncirus* associated with apomixis. Though codominant marker types such as RFLP, SSR, SCAR, CAPS, and SNPs were being used earlier, the numbers of such loci available were severely limited. Consequently, there has been a limited ability to interrelate maps and/or markers developed in different populations or targeting different QTLs, and there were very few chromosome-specific anchor markers that could enable comparative mapping efforts between different genetic resources of citrus. The processes for developing such markers previously were very time and labor intensive, and the efficiencies were extremely low. Further, some of these markers were developed in the absence of genome or transcriptome sequences, and as such they might be considered to be gene anonymous. However, the revolution in sequencing technologies, including the sequencing of BAC clones and fairly extensive EST libraries for citrus accessions under multiple conditions, has produced a very substantial resource for high-throughput development, verification, and utilization of molecular markers for citrus linkage mapping, which meets the desired criteria. These markers frequently are based on EST sequences and therefore represent specific genes, which functions may be known or estimated in some cases. For example, Omura et al. [25, 26] first reported development of 131 mapped CAPS markers derived from EST sequences. Using a backcross citrus population, these markers were assigned into nine linkage groups that accounted for 685 cM coverage, and they were found to be portable to another population. With the rapid increase in publicly available EST databases in the USA [27] and publicly available software programs, large-scale searches for various types of SSR motifs and efficient design of appropriate primers have made it possible to identify and map EST-SSRs in citrus [21, 28]. The first such map for sweet orange and *P. trifoliata* was published in 2007 [21], and it is being expanded collaboratively [29]. New international, collaborative EST-SSR mapping efforts are currently underway [30] using other citrus-based families as part of a plan intended to lead to the full-length sequence of a haploid citrus genome, to be integrated with physical and genetic maps based on BAC end sequencing, high-throughput marker saturation, and mapping traits of economic importance to genetic improvement of citrus. These extensive international efforts are being promoted and coordinated by the International Citrus Genome Consortium (ICGC), currently chaired by F. G. Gmitter of the University of Florida, USA. New technology will continue to enhance progress toward high-resolution and highly informative maps of citrus genomes in the future. Currently, efforts are underway in several labs around the world to utilize microarrays for mapping SNPs in various families and genetic backgrounds, and, as a full genome sequence comes forward for citrus, followed by additional resequencing of other genomes of interest, the genomic identification and locations of thousands of trait-relevant SNPs will become known and exploited for genetic improvement of the crop.

## 3. PHYSICAL MAPPING

The main challenge for a comprehensive and meaningful description of the genomes is the integration of the DNA marker-based genetic maps with physical maps, and eventually with DNA sequence of the whole genome, the ultimate physical map. Large genomic DNA insert-containing libraries are required for physical mapping, positional cloning, and genome sequencing of complex genomes. The physical mapping of complex genomes is based on the construction of a genomic library, and the determination of the overlaps between the inserts of the mapping clones in order to generate an ordered, cloned representation of nearly all the sequences present in the target genome.

For the generation of high-resolution physical maps, the construction of bacterial artificial chromosome (BAC) libraries containing clones with large DNA fragments appears to be indispensable. The BAC cloning system has become a dominant system over others to clone large genomic DNA inserts. BAC clone collections and BAC-based contig maps are indeed powerful tools having multiple applications in genomics such as supporting positional cloning or to aid large-scale assembly of whole genomes. In whole genome sequencing projects, BAC end sequences (BES, paired-end reads) are also of inestimable help for the integration of the physical map with the genome sequence. Furthermore, in many agricultural important species, BAC clones and physical maps are being rapidly developed since they are essential components in linking phenotypic traits to the responsible genetic variation, to integrate the genetic data, for the comparative analysis of genomes, and to speed up and improve potential and effectiveness of marker-assisted selection (MAS) for breeding.

### 3.1. BAC libraries

In citrus, Yang et al. [31] and Deng et al. [32] independently constructed two BAC libraries as part of a map-based, or positional, cloning strategy with the idea of identifying BAC clones spanning the genetic region identified as containing gene(s) for resistance to CTV. CTV is the causal agent of several diseases causing significant economic damage and losses to citrus worldwide. Broad spectrum resistance to CTV was previously associated with a single dominant gene, *Ctv*, characterized in *P. trifoliata*, a sexually compatible relative of citrus [23]. In order to clone this gene, Yang et al. [31] constructed a BAC library from an individual plant homozygous for *Ctv*. The library contained 45 000 clones with an average insert size of 80 kb. *Ctv* was initially mapped to a 282-kb region including a disease resistance gene cluster with seven members and eight retrotransposons clustered [33]. Sequence analysis of the *Ctv* surrounding genomic region located the locus into a 121-kb *Poncirus* region comprising 10 genes. All 10 genes were individually cloned in *Agrobacterium*-based binary vector and used to transform susceptible varieties [34] to test their resistance capability.

In a parallel effort, a BAC library was constructed from the genomic DNA of an intergeneric *Citrus* and *Poncirus* hybrid for molecular isolation of disease resistance genes, including *Ctv* [32]. The library of 24 000 clones with an average insert size of 115 kb was screened with DNA markers linked to the *Ctv* gene and citrus disease resistance gene candidate (RGC) sequences. A few clones were isolated with each of the CTV resistance gene-linked markers, and several hundred others were identified using previously cloned citrus RGC sequences as probes [35]. Further fingerprinting and assembly resulted in the identification of 25 contigs of 120–250 kb. Additional libraries were developed from the same intergeneric hybrid for the purpose of map-based cloning of *Ctv*. From these libraries, full contigs were constructed that spanned both the resistance allele from *Poncirus* and the susceptibility allele from the *Citrus* chromosome. These clones were fully sequenced and assembled. Comparisons of the resistance and susceptibility allelic genomic sequences revealed that the levels of similarity varied from region to region. Within the region where the most likely *Ctv* candidate genes were delimited, based on fine genetic mapping and predicted by various sequence analysis programs, there were 2 NBS-LRR candidate genes found in *Ctv* that were completely missing from the *Ctv* sequence. Based on the sequence analysis and the fine mapping results, it was concluded that either one or both of these unique sequences should be considered the first priority candidates for *Ctv*.

A further exploitation of this BAC library resulted in identification of other disease resistance gene-like DNA sequences, using a PCR approach with degenerate primers designed from conserved NBS (nucleotide-binding site) motifs [35]. In addition to the three amplified DNA fragment markers associated with the citrus tristeza virus resistance gene (*Ctv*), another fragment (Pt8a) was found to be associated with the major gene responsible for the citrus nematode resistance (*Tyr1*). In a similar approach, degenerate primers for the conserved motifs in the kinase domains of the plant disease resistant genes (R) of rice *Xa21* and tomato *Pto* were used in PCR amplification to identify resistance gene candidates. Twenty-nine sequences highly similar to the kinase domain of *Xa21* were cloned and characterized [36]). Using the BAC library, two full-length sequences, including upstream promoters and downstream terminating sequences, were identified. Markers derived from these *Xa21*-like sequences have been found linked to putative QTLs for citrus canker resistance segregating among hybrids derived from *Citrus ichangensis* Swing. with *Citrus limettoides* Tan. (Gmitter, unpublished data).

### 3.2. Physical mapping

Two communications at the PAG XV meeting in San Diego, 2007, reported progress on the construction of physical maps of citrus [37, 38]. The Spanish Citrus Genomic Consortium has constructed three BAC libraries from Clementine mandarin (EcoR I, Hind III, and MboI) containing a total of 57 000 clones with an average insert size of 120 kb (19x coverage). Half of these BAC clones were end-sequenced (29 Mb), and these sequences analyzed [37]. The sequence analysis revealed that most abundant known retroelements were LTR elements, especially Ty-1 Copia [39] and Gypsy [40] elements, while known DNA transposons were scarce. Basic local alignment search tool (BLAST) searches also identified about 14 000 clones with coding regions in a least one end and therefore putative euchromatin regions. BAC end sequences were also searched for single nucleotide polymorphisms (SNPs) and simple sequence repeats (SSRs) in the Clementine genome. These initial analyses identified more than 2800 sequence repeats in coding regions out of 7700 putative SSRs. Some 1.7% of the reads had high similarity with the sequence of the *Citrus sinensis* chloroplast genome [41], suggesting that about 70% of the chloroplast genome of Clementine was similarly recovered in this sequencing effort. In parallel, a physical map derived from the same 28 000-clone set of the Clementine BAC libraries is being constructed by restriction enzyme fragment fingerprinting, and work is in progress to place on it a sufficient number of genetically mapped markers to anchor and orientate the contigs (Ollitrault, personal communication). It is expected that the paired-end reads will also aid integration of the genetic and physical maps.

The Citrus Genome Analysis Team from Japan has also communicated the construction of a physical map of citrus by high-information-content fingerprinting (HICF) analysis of a BAC library from Satsuma mandarin (*Citrus unshiu* Marc.) consisting of 37 000 clones, with 13.3x of citrus genome [38]. More than 6000 BAC clones from the library were fluorescent labeled using the SNaPshot kit after digestion with BamHI, EcoRI, XbaI, and XhoI and assembly of BAC fingerprints by FPC resulted in approximately 1000 contigs (1.6x coverage). Consistent assembly among contigs obtained by fingerprint analysis and physical maps obtained by BAC walking with both molecular markers and BAC end sequences was observed. Further evaluations by additional clones, assignment of molecular markers for the contigs, and gap filling by BAC end sequencing to complete the physical map were also reported to be in progress [38].

A BAC library of Ridge Pineapple sweet orange was produced by Michael Bausher (USDA-ARS, Ft. Pierce, FL, USA) containing 18 432 clones (BamHI/Mbo I) with an average insert size of 145 kb, or an estimated 7x coverage. A total of 16 727 clones from this library have been fingerprinted and assembled into 472 contigs, as of August 2006. Access is freely available to the public at http://phymap.ucdavis.edu:8080/citrus. This resource was searched by EST-SSR overgo probes to identify BAC clones in six known heterozygous genomic regions containing polymorphic alleles of mandarin and/or pummelo ancestry (the putative ancestral genomic contributors to sweet orange). These BAC clones were then sequenced and assembled “blind” to assess the difficulties in assembling sequences of the heterozygous sweet orange.

The goal of genome-wide integrated physical and genetic maps is a priority in citrus genomics since it will provide the essential and powerful tools for research into the citrus genome, such as effective positional cloning, marker development, high-throughput EST mapping, and large-scale genome sequencing and assembly.

## 4. CITRUS SEQUENCING

One of the major goals of the International Citrus Genomics Consortium is to provide a high quality sequence of a citrus genome. Sequencing of the citrus genome will facilitate the comparison of herbaceous and woody perennial genomes and provide a valuable resource for studying significant biological questions of critical importance to genetic improvement of citrus. From a scientific perspective, citrus as a fruit tree developing nonclimacteric fruits possesses a combination of interesting biological characteristics such as apomixis, gametophytic self- and cross-incompatibility, juvenility, deciduousness/evergreen foliage, dormancy, seasonality, root/shoot interaction, oil glands, nutraceutical compounds, and plant-pathogen interactions. Additionally, citrus is the most economically significant fruit crop produced in the world, although citrus production is severely threatened by pest, disease, and environmental problems to which current commercial rootstock and scion cultivars are susceptible.

In February 2004, a proposal prepared and supported by the National Citrus Genomics Steering Committee (USA) and the International Citrus Genomics Consortium (ICGC, composed of researchers from Australia, Brazil, China, France, Israel, Italy, Japan, Spain, and USA) to sequence the genome of sweet orange was presented to the Joint Genome Institute (JGI). This institution reported at the beginning of 2007 to have produced a low coverage (ca. 1.2x) whole-genome shotgun sequence of *Citrus sinensis* (sweet orange) by sequencing ends of about 126 000 fosmid clones containing 40 kb inserts and 257 000 plasmid clones containing 8 kb inserts [42]. Total sequence coverage, available at http://harvest.ucr.edu/, was about 473 Mb, coverage apparently insufficient to provide quality assembling of the high heterozygous sweet orange.

In January 2007, the Steering Committee of the ICGC met at JGI in California, USA to reassess the present status of citrus genome research and to forge plans for future collaborative efforts. The first outcome of the meeting was a decision to shift focus to a haploid genome as the target for sequencing, rather than the previously stated target of sweet orange; this consideration was made to eliminate the difficulties associated with quality assembly of highly heterozygous diploid sweet orange genome. A haploid derived genome sequence should serve as the highest quality reference genome for all future genomic research efforts. It was required that the haploid (or di- or tri-haploid individual chosen) should be available for free distribution internationally, and that it should be pathogen-free, exhibit robust vegetative growth (as a partial guarantee against gross genome defects), and be relatively easy to maintain. Teams were established to verify chromosome number and to assess candidates for homozygosity using SSR markers representing good genome coverage based on linkage maps. Additionally, the candidates will be assessed using at least two available citrus microarray platforms in an effort to insure that there are no large deletions or other cytogenetic defects. Currently there are three candidates, all derived from Clementine mandarin, that are being evaluated according to this plan. It is envisaged that the international collaboration will be supported from various agencies and sources in different partner nations, that the sequence information will be quickly deposited and shared freely among the participating laboratories, and that the goal will be achieved once there is 8 to 10x coverage. Currently, there are funding commitments from USA, Spain, France, Italy, and China.

The complete chloroplast genome sequence of *Citrus sinensis* was recently provided by Bausher et al. [41]. It is 160,129 bp in length and contains 133 genes (89 protein-coding, 4 rRNAs, and 30 distinct tRNAs). The genome included 29 direct and inverted repeats 30 bp or longer, and comparison of protein-coding sequences with expressed sequence tags revealed six putative RNA edits. Phylogenetic analyses provide strong support for the monophyly of eurosids II and for the placement of Citrus (Sapindales) sister to a clade including the Malvales/Brassicales.

## 5. FUNCTIONAL GENOMICS

### 5.1. EST sequencing

The first sets of ESTs (expressed sequence tags) from any citrus material came from the pioneering work of Omura and coworkers who reported about 3000 partial sequences of cDNA clones from libraries derived from seeds, and from developing and mature fruit and albedo tissue, during the second half of the 1990s [43]. Later, a set of 6500 ESTs derived from whole seedlings of sweet orange was developed by Bausher et al. [44], and a new contribution of 600 sequences from *Citrus unshiu* of the Japanese team was also reported [45]. Since then, various groups (including Roose and Close at University of California at Riverside [UCR], Dandekar at University of California at Davis [UCD], and the Spanish Citrus Genomics Consortium at Valencia) have contributed to EST sequencing efforts using several species, mostly *C. sinensis* (sweet orange), *C. clementina* (Clementine mandarin), *C. paradisi* (grapefruit), *Poncirus trifoliata,* and other hybrids (*C. sinensis* × *Poncirus trifoliata*, Carrizo citrange). The total resource has reached 232 808 citrus sequences in the National Center for Biotechnology Information (NCBI) EST database as of May 2007. This EST collection includes a wide representation of sequences from many cDNA libraries derived from multiple reproductive (flowers, ovaries, fruits, seeds) and vegetative (roots, leaves, buds) organs and tissues (pulp flesh, flavedo, abscission zones) at different developmental stages and challenged with biotic (*Phytophthora*, citrus tristeza virus, herbivory, *Penicillium*) and abiotic (salinity, iron deficiency, water deficit) agents, and elicitor and hormonal treatments.

Although a compressive analysis of all ESTs in public databases has not been performed, several subsets of the data have been partially analyzed. Forment et al. [46], for example, generated 25 cDNA libraries covering different conditions and from 22 635 high-quality ESTs identified 11 836 putative unigenes. A third of these unique sequences was reported not to have Arabidopsis orthologues. From a deeper analysis of a collection of 54 000 single-pass ESTs, derived mostly from a normalized full-length cDNA library (41 000 ESTs) and nine additional standard libraries representing particular treatments and tissues from several selected varieties and rootstocks, Terol et al. [47] identified 13 000 putative unigenes with significant BLAST hits. Further analyses and comparisons with *Arabidopsis* suggested the occurrence of citrus paralogues, putative conserved orthologues, single copy genes, duplication events, and increased number of genes for specific pathways. Interestingly, the sequences of the genes belonging to these different species were essentially identical, suggesting that their differential behavior cannot be attributed to major sequence divergences. Nearly 17% (2250 total) of the predicted citrus unigenes had no detectable similarity to *Arabidopsis* genes, and of these, 647 unigenes produced significant hits only to *Citrus* species, suggesting that these clusters might be putative *Citrus* exclusive genes [47]. This work also contributed over 8500 clones carrying putative full-length cDNA sequences. Full-length sequences and clones that are valuable tools since they can facilitate proper prediction of gene structures provide a useful resource for functional analysis and may greatly facilitate annotation of the full genome sequence. BLAST searches against sequenced citrus ESTs are possible through several open database projects (i.e., http://harvest.ucr.edu/; http://cgf.ucdavis.edu/; http://bioinfo.ibmcp.upv.es/genomics/cfgpDB/) or data deposited in GenBank. Although predictions from EST clustering tend to overestimate the total number of genes, these citrus EST sequences are apparently derived from perhaps 40 to 50 000 genes, a number more similar to that reported in *Populus* than that in *Arabidopsis*.

In addition, Machado et al. [48] reported at the PAG-XV meeting that Brazilian researchers have developed a huge EST sequencing effort. According to this communication, the CitEST (http://citest.centrodecitricultura.br/) Brazilian database including more than 260 000 valid reads contained unigene sets from several citrus species but mainly sweet orange, mandarin, and *Poncirus trifoliata*. These ESTs were generated from several libraries under biotic (*Xylella fastidiosa*, CTV, Citrus Leprosis Virus, *Phytophthora*, mite) and abiotic (drought) stresses, and during fruit development.

### 5.2. Microarrays platforms

The importance of microarray technology for transcript profiling approaches in functional genomics is increasing exponentially in practically all plant systems and, in particular, in many agricultural crops. In citrus, the first transcript profiling data was reported by Shimada et al. [49] who constructed a cDNA microarray to monitor expression of mRNA from 2213 genes during fruit development. Since then, several citrus DNA microarray platforms were developed. The Spanish Citrus Genomic Consortium developed a first generation cDNA microarray containing 12 672 probes corresponding to 6875 putative unigenes of a 22 000-EST collection [46]. Subsequently, a second-generation microarray comprising 12 000 unigenes was released and, shortly afterwards, the Consortium produced the current version, a higher density citrus microarray composed of 24 000-element cDNA array containing 20 000 unigenes, based on nearly 90 000 high-quality sequences generated from 52 different cDNA libraries.

Similarly, a citrus 22 K oligoarray containing 21 495 independent ESTs from Citrus species has been recently developed in Japan. The information regarding this platform is available at the website http://www.fruit.affrc.go.jp/index-e.html. This tool has already produced very useful information [50]. In 2006, Affymetrix developed and released a citrus GeneChip containing 960 444 total 25-mer oligos in an 11 micron format (http://www.affymetrix.com/analysis/index.affx), this product came through close collaboration with Close and Roose (UCR) and was based on the NCBI citrus EST collection that was available at the time of its design. About two-third of the content was designed for gene expression analysis using 30 264 probe sets. Most of the remaining one-third was designed to genotype 3219 genes using 5023 SNPs identified in ESTs from *Citrus sinensis* and other citrus species. The citrus chip also contained probe sets for detection of several pathogens and commonly used transgenes and a representation of the region of the *P. trifoliata* genome containing *Ctv*, the CTV resistance allele. In January 2007 at PAG, it was communicated that the GeneChip Citrus Genome Array is being used in Israel, for example, to analyze transcription profiles of bud sprouting as related to alternate bearing behavior [51]. Additional work is currently underway in various labs using the Affymetrix product for high-throughput linkage mapping, and to assess gene expression under various physiological and pathogen and/or pest challenges, it is anticipated that there will be several reports published within the next year on these research projects.

Several other communications presented at PAG XV, for instance, Deng et al. [52] and elsewhere reported other research projects using cDNA citrus microarrays or smaller custom arrays based on subtractive libraries. Some of them include analysis of transcriptional responses of 1731 genes to herbivory [53], of 312 subtracted genes in abscission zones (Tadeo and Talon, unpublished data), and the investigation of citrus canker resistance in kumquat (*Fortunella spp*) using an array with 2254 elements [54].

### 5.3. Gene expression and transcriptome profiling

In a recent review, Jansson and Douglas [55] explained the usefulness of *Populus* as a new plant system model offering new insights in many physiological processes that cannot be easily studied in *Arabidopsis* or rice, the two main models for plant biology. The strength of this proposition, sustained by the completion of the whole poplar genome sequence and the development of several genetics and genomics tools, holds promise to elucidate major tree-specific traits such as wood formation, long-term perennial growth, biotic interactions, and others. The availability of a second tree model can provide contrasting data on plant genome evolution, gene family structure, and other pivotal tree traits. Citrus as a fruit tree not only will promote achievement of these goals but more importantly offers a suitable system to study “fruit growth and quality,” a fundamental plant trait for which *Arabidopsis*, rice, and poplar are not useful systems. Although these model plant systems, including tomato, are crucial to understand plant growth and development, the dramatic developmental differences found across species are channeling many efforts to genomic and post-genomic studies of crop plant species, rather than retaining focus solely on these model species.

Citrus possesses an enormous unexplored potential to reveal relevant plant growth processes and some responses that probably cannot be studied in any other plant. Although functional genomics in citrus is currently in its infancy, the particular citrus biology suggests that citrus may contain a reservoir of genes with peculiar and unique functions. One of the first steps to assign functions to unknown genes is the large-scale gene analysis of the transcriptome. In citrus, before microarray availability, gene expression in developmental and environmentally regulated processes, as in many other systems, was mostly studied through differential display techniques (i.e., DDRT-PCR). Genes involved in many processes were also identified after subtractive hybridization of cDNA libraries constructed from two different conditions. The main targets in citrus research have been those physiological processes that sustain major commercial traits. Below, we summarize the knowledge gained in these several areas.

#### 5.3.1. Fruit growth and ripening

While in tomato (a climacteric fruit) great strides have been made in the areas of ethylene regulation, carotenoid accumulation, and cell wall metabolism, in nonclimacteric citrus fruit the general information is substantially less. Mature citrus fruits release low amounts of ethylene but respond to exogenous ethylene by accelerating respiration, chlorophyll degradation, and carotenoid deposition. In these fruits, very low rates of ethylene production have been associated with constitutive expression of the 1-aminocyclopropane-1-carboxylate synthase 2 (CsACS2) and ethylene receptor CsETR1 genes, indicating that citrus possesses a system I machinery. However, it has been reported that a climacteric-like rise in ethylene production, preceded by induction of the genes for CsACS1, ACC oxidase1, and the ethylene receptor CsERS1, characteristic of a system II-like, appears to be present in young fruitlets [56]. It is well known that ethylene accelerates the molecular changes in the carotenoid biosynthesis naturally occurring during maturation (see below), while gibberellins and nitrates, two ripening retardants, reduce expression of early carotenoid biosynthetic genes and repress pheophorbide a oxygenase (PaO) expression [57, 58], a gene involved in chlorophyll disappearance. Other characteristic genes induced by ethylene are ferredoxins or *thi,* a gene involved in thiamine biosynthesis. The large ABA amounts found in the peel of citrus fruit during maturation appear to be synthesized by two 9-cis-epoxycarotenoid dioxygenases (NCED) with differential spatial and environmental expression [59, 60].

In citrus, an initial small-scale EST sequencing project from mature fruit resulted in the identification of 20% of the sequences as encoding for metallothionein [61]. The abundance of these kinds of genes was confirmed later in more developed citrus arrays. Later, Shimada et al. [49] used a citrus cDNA microarray containing 2213 independent genes to examine gene expression during fruit development and reported that the expression profile in the different tissues of the fruit, flesh, albedo, and flavedo was rather different. Recently, a comprehensive transcriptome analysis using a citrus 22 K oligoarray was performed to identify ethylene-responsive genes in mandarin fruit [50]. In the 72 hours after ethylene treatment, 1493 genes were shown to be modulated by the hormone. Ethylene repressed the transcription of most genes involved in photosynthesis, chloroplast biogenesis, and sugar metabolism, while it induced the transcription of several genes related to resistance, defense, stress, amino acid synthesis, protein degradation, and secondary metabolism. The sensitivity and responsive patterns to exogenous ethylene were significantly different among carotenoid biosynthesis genes (see below). Furthermore, most of the ethylene biosynthesis genes and its signal transduction components did not show any significant expression change after ethylene treatment. Interestingly, a type II ethylene receptor (ETR2) showed higher sensitivity to exogenous ethylene than two other type I ethylene receptors (*CsETR1* and *CsERS1*), suggesting that ETR2 might be associated with low ethylene sensitivity in mature fruit [50].

During the last decades, research on citrus fruit flavor that depends upon multiple compounds, mostly sugars, acids, and flavanones, has received considerable attention because of both the uniqueness of the physiological processes sustaining this trait and the potential importance of these components to human health. To date, the most comprehensive study on the transcriptome profiling of the citrus fruit flesh was presented by Cercós et al. [62] who examined gene expression with the first generation Spanish cDNA microarray during development and ripening of self-incompatible *Citrus clementina*. They reported that as many as 2243 putative unigenes showed significant expression changes while functional classification revealed that genes encoding for regulatory proteins were most significantly overrepresented approximately within the middle of the rapid fruit growth phase; this suggested that fruits at this stage were reprogramming developmental commands to face the complex cellular modifications during ripening. Most pivotal changes were related to carbohydrate build up, acid reduction, modifications in secondary metabolism, carotenoid accumulation, and chlorophyll decreases. Alterations of the transcriptome associated with carbon accumulation were expected since it was known that expression of several homologues of pivotal genes implicated in carbon metabolism (e.g., phosphoenolpyruvate carboxykinase, ADP-glucose pyrophosporylase, sucrose synthase, and sucrose phosphatase synthase) and transport (i.e., sucrose transporters) during fruit growth considerably changed [63, 64]. In general, these genes appear to belong to small families including few members, showing differential spatial and temporal expression.

On the other hand, developing citrus fruits accumulate a considerable amount of citric acid in the vacuoles of the juice sac cells, although before ripening this high concentration is considerably reduced. The rate of change and final acid levels are perceived as major components for citrus fruit quality. Research in gene regulation of acid metabolism, however, has not led to a full understanding of this essential process. There is considerable evidence, nevertheless, obtained comparing acidless and acidic varieties that activity and expression of citrate synthase were not responsible for these differences [65]. Another gene characterized in citrus was NADP(+)-isocitrate dehydrogenase (NADP-IDH), encoding for an enzyme involved in citrate metabolism. Recently, a citrate transporter gene has been reported encoding a novel vacuolar citrate/symporter that is able to mediate citrate vacuolar efflux through the electroneutral cotransport of H+ and citrate ions [66]. Interestingly, the transcriptomic study together with the analyses of selected metabolites suggested the occurrence of specific metabolic alternatives during citric acid catabolism [62]. Microarray data suggested that citrate was sequentially metabolized to glutamate that was finally catabolized through the gamma-aminobutyrate (GABA) shunt. This observation was of special relevance since it linked an efficient major proton-consuming reaction with high acid levels. This work provides a convincing explanation for the strong reduction of both citrate and cytoplasmic acidity that takes place in citrus fruit flesh during development and ripening.

Transcript profiling also revealed down-regulation patterns of gene expression for anthocyanin and flavonoid biosynthesis, confirming previous observations. Thus, it was known that in common oranges there was a differential repression of some of the enzymes of anthocyanin biosynthetic pathway, namely chalcone synthase (CHS), anthocyanidin synthase (ANS), and UDP-glucose-flavonoid 3-O-glucosyltransferase (UFGT) [67], in contrast to “blood” pigmented oranges. However, anthocyanin and gene expression associated with anthocyanin synthesis increased at low temperature [68]. Flavanones, a flavonoid subgroup, that greatly contribute to the bitter flavor of grapefruit and other citrus, have also been the subject of intensive work and pivotal genes of this biosynthetic pathway such as CHS, chalcone isomerase (CHI), flavanone 3-hydroxylase (F3H), dihydroflavonol 4-reductase (DFR), and flavonol synthase (FLS) have been isolated and characterized [69, 70]. In an elegant work, Frydman et al. [71] demonstrated that the key flavor-determining step of citrus flavanone biosynthesis was catalyzed by rhamnosyltransferases. They demonstrated that 1,2 rhamnosyltransferases catalyzed biosynthesis of the bitter neohesperidosides, while 1,6 rhamnosyltransferases catalyzed biosynthesis of the tasteless rutinosides. Bitter species, such as grapefruit and pummelo, accumulated bitter flavanone-7-O-neohesperidosides (naringin, the major flavonoid glycoside in grapefruit) responsible, in part, for their characteristic juice flavor, while nonbitter species, such as mandarin and orange, accumulated only tasteless flavanone-7-O-rutinosides.

Bitterness in citrus also is associated with the presence of limonoids, triterpene derivatives that confer the scent to fresh lemon and oranges. Kita et al. [72] isolated a cDNA clone encoding limonoid UDP-glucosyltransferase (limonoid GTase) that regulated the conversion of limonoid aglycones such as limonin, a bitter compound, to their nonbitter glucosides.

In addition to limonoids, citrus fruits possess unique aromas rarely found in other fruit species produced by other terpenes. This is also an area of high research interest. Monoterpenes (*d*-limonene terpinene and pinene) and other low-abundance sesquiterpenes (valencene, nootkatone, and *α*- and *β*-sinensal) stand out in citrus as important aroma and also flavor compounds. Lücker et al. [73] and Shimada et al. [74] isolated various monoterpene synthases (*d*-limonene, *γ*-terpinene, *β*-pinene synthase, *β*-ocimene, and cineole synthase), and it has also been shown that their metabolic engineering produced new aromas in tobacco [75]. Monoterpene synthesis takes place in epithelial cells surrounding the secretory cavities that contain the oil glands in the flavedo [76]. Regarding sesquiterpene production, Sharon-Asa et al. [77] identified a sesquiterpene synthase-encoding gene, regulating the conversion of farnesyl diphosphate to a single sesquiterpene, valencene. They reported the transcript that was responsive to ethylene naturally accumulated only towards fruit maturation. Other putative sesquiterpene synthases, such as *β*-farnesene synthase, have also been cloned.

Work is also in progress to characterize induced mutants that exhibit altered fragrance (*alf*) and abnormal number of oil glands in the flavedo [78]. Transcriptome analysis of fruits from these mutants showed changes in expression profile of genes encoding enzymes involved in the biosynthesis of volatile compounds derived from isoprenoid and phenylpropanoid pathways. In fruits of *alf*, several genes with different biological functions were down-regulated although genes coding for a new putative terpene synthase (TPS) and an O-methyltransferase (OMT), apparently involved in secondary metabolism of volatile compounds, had the highest differences in expression. In the mutant with lower number of glands, transcript profiling also revealed strong down-regulation of genes encoding enzymes from the phenylpropanoid and isoprenoid biosynthetic pathways. In a similar approach, Ishikawa et al. [79] used a 22 K citrus microarray to analyze gene expression in a mutant that shows smoother rind and decreased numbers of oil glands. The authors reported that the genes of the nonmevalonate pathway of isoprenoid synthesis and monoterpene synthases were down-regulated in the mutant.

Tetraterpenes are also crucial components of citrus fruit that contains one of the greatest arrays of carotenoids found in any plant. The simultaneous carotenoid accumulation and chlorophyll reduction occurring during natural ripening indeed determines the color of the fruit peel, a most valuable characteristic of perceived fruit quality. Many pivotal genes of the carotenoid pathway have been cloned in citrus (phytoene synthase (CitPSY), phytoene desaturase (CitPDS), *ζ*-carotene (car) desaturase (CitZDS), carotenoid isomerase (CitCRTISO), lycopene *β*-cyclase (CitLCYb), *β*-ring hydroxylase (CitHYb), zeaxanthin (CitZEA) epoxidase (CitZEP), lycopene *β*-cyclase (CitLCYb), and lycopene *ε*-cyclase (CitLCYe), and their expression has been correlated with the accumulation of carotenoids in fruit [80, 81]. It was reported that the transition of peel color from green to orange, and the change from *β*,*ε*-carotenoid to *β*,*β*-carotenoid accumulation was accompanied by the disappearance of CitLCYe and the increase in CitLCYb transcripts. As fruit maturation progressed, a concomitant increase in the expression of CitPSY, CitPDS, CitZDS, CitLCYb, CitHYb, and CitZEP led to massive *β*,*β*-xanthophyll accumulation. Cercós et al. [62] showed that expression of carotenoid biosynthetic genes in fruit flesh followed rather similar changes. Mutations of flesh color are being investigated in China using a citrus cDNA array with 6000 unigenes [52]. 

In contrast to carotenoid accumulation, there have been fewer studies of the chlorophyll degradation processes in citrus. Previous work on the regulation of catabolism showed that chlorophyllase (*CLH*) was constitutively expressed during natural fruit development [82]. Recent results suggest that *CLH* functions as a rate-limiting enzyme in chlorophyllcatabolism controlled via post-translational regulation [83]. It is also known that pheophorbide a oxygenase (*PaO*) and geranylgeranyl reductase expression, correlated with chlorophyll degradation [57]. Recent work upon “nan,” a stay-green mutant of Navel orange that produces fruit with abnormal brown flavedo, showed that typical ripening-related chlorophyll (Chl) degradation was impaired in this mutant. Transcript and proteomic profilings revealed that a citrus orthologue of a number of *SGR* (*stay green*) genes was expressed at substantially lower levels in “nan” both prior to and during ripening [84]. The “*nan*” mutation also resulted in the suppressed expression of numerous photosynthesis-related genes and in the induction of genes associated with oxidative stress. The transcriptome of other selected citrus mutants is also being investigated to identify gene functions related to fruit quality that in citrus are barely accessible through genetic approaches. To this end, three collections of induced mutated lines (EMS, gamma rays and fast neutrons) have been generated, comprising 10 000 potential [84] mutants.

With the exception of thermostable pectin methylesterase activity [85, 86] that greatly reduces citrus juice quality, cell wall metabolism in citrus has been studied as related to fruit abscission, a major component of final yield. One of the strategies for the identification of abscission-related genes followed by Dr. Burns' team (University of Florida) was based upon the isolation of ethylene-induced genes in the calyx, the laminar, and the floral abscission zones. The role of ethylene on the regulation of abscission has been widely illustrated for decades, and several works have shown that ethylene is the primary effector activating the abscission pathway in citrus [87, 88]. Differential display and subtractive cDNA library screening were also used to search for abscission-related metabolism changes. Important components of the citrus abscission process were thus associated with expression and/or activity of pivotal enzymes of cell wall metabolism (glucanases, polygalacturonases, galactosidases, and other hydrolases; [89, 90]), hormonal synthesis, and signal transduction (i.e., ACC synthases and oxydases) and secondary metabolism/PR proteins (i.e., phenylalanine ammonia lyase, chitinases). Yuan et al. [91] also demonstrated that differential expression of ACC synthase 1 and ACC oxidase genes was associated with reduction of ethephon-enhanced leaf abscission by guanfacine, a G-protein-coupled alpha-(2A)-adrenoreceptor selective antagonist, and suggested a link between G-protein-related signalling and abscission. Interestingly, guanfacine had little effect on ethephon-enhanced fruit loosening. In spite of this information, major regulators of the abscission process in citrus are still mostly unknown although both custom manufactured and large-scale microarrays, in some instances, coupled to laser assisted microdissection (LAM) are currently being used in Florida and Spain to gain new insight into this process. Part of these transcriptomic profiling studies has been summarized in a recent Ph.D. dissertation presenting a model of leaf abscission events occurring at the laminar abscission zone [60]. The two-stage model proposes a first phase of activation, mostly characterized by the activation of signalling pathways (hormones, phospholipids, calcium, and oxygen reactive species). In a second stage, the execution phase, degradation of the cell wall by hydrolytic enzymes would be culminated and sugar-nucleotide metabolism for cell elongation induced. The process would end with the promotion of a double defensive program intended to protect the living zone remaining attached to the plant including deposition of physical barriers (callose and lignin) and induction of pathogen resistance.

Several other microarray studies on citrus growth and ripening are under development and have not been published yet. For instance, Dr. Sadka is investigating with the GeneChip Citrus Genome Array (Affymetrix) the transcriptome modifications occurring during the induction of flower bud differentiation using “on” and “off” trees [51], taking advantage of the alternate bearing behavior, a process regulating differentially carbohydrate-related gene expression [92]. In Brazil, the sequencing carried out at the Centro APTA Citros “Sylvio Moreira”—IAC (Brazil) that has generated one of the most important databases for this genus in the world is being extensively used to produce “*in silico*” analyses. This approach is yielding information not only related to fruit growth and development (terpene production, cell wall metabolism, etc.) but also in the biotic stress field [48].

#### 5.3.2. Responses to pathogenic and environmental stresses

In citrus, gene expression associated with the responses to biotic and abiotic stresses has targeted a limited number of genes in spite of the economical importance of the citrus diseases and environmental constrains. Multiple pathogens provoke a range of citrus disorders, mostly fungal (leaf spot, *Alternaria*; mold, *Penicillium*; post-bloom fruit drop, *Colletotrichum acutatum*; root rot, *Phytophthora*), bacterial (canker, *Xanthomonas axonopodis*; citrus variegated chlorosis, *Xylella fastidiosa*; Huanglongbing or greening, *Candidatus* Liberibacter), and viral diseases (citrus tristeza virus, CTV; citrus leprosis virus, CiLV). Environmental stresses include cold temperatures, drought, flooding, salinity, and high and low soil pH, among others.

In response to the inoculation with conidia of *Alternaria*, at least two cytosolic antifungal miraculins with protease inhibitor activity were strongly up regulated. Actually, induction of miraculin expression is one of the most prominent responses observed in microarray experiments performed in open field experiments. Both miraculin genes responded to methyl jasmonate and were antagonized by salicylate [93]. Several studies with the green mold pathogen, *Penicillium digitatum*, also indicated that genes such as thioredoxins, the *gnsl* gene (beta-1,3-endoglucanase activity), and chitinases are major components of the molecular mechanisms involved in activation of pathogen defense in citrus. Other responsive genes reported in citrus were epoxide hydrolase and hidroperoxide lyase. It has also been shown that the fungus *Colletotrichum acutatum* altered hormonal homeostasis increasing both levels of ethylene, indole-3-acetic acid, cis-jasmonic acid (JA) and salicylic acid (SA), and associated gene expression [94].

Gandía et al. [95] have recently presented data on the transcriptional response of citrus to infection with severe and mild isolates of citrus tristeza virus. These studies concluded that gene expression was only significantly altered with the severe isolate. Changes detected in the citrus transcriptome after infection with this isolate were predominantly associated with symptom expression (chlorophyllases, SAM transferases, ACC oxidase, and lipid transfer proteins), defense mechanism, and general responses to stress (miraculins, superoxide dismutases, glutation transferees, NBS-LRR resistance genes, thioredoxin, protease inhibitors, ubiquitin ligases, etc.).

To study the mechanisms of canker resistance in kumquat, a custom microarray using 2254 ESTs from subtractive libraries is being utilized to determine the response to infective bacteria in an incompatible interaction [54]. The macroscopic phenotype, a delayed hypersensitive response in the inoculated leaves, was accompanied by altered expression of 1245 genes. This study identified major components of the incompatible interaction, reactive oxygen species (ROS) production, and programmed cell death (PCD). In addition, a number of common defense mechanisms besides a number of resistance genes and putative receptors were also identified.

Citrus plants are also very liable to infestation by aphids, whitefly, and other insects as well as being susceptible to herbivory. Mozoruk et al. [53] described how nylon filter cDNA arrays were used to analyze the transcriptional changes of 1731 citrus unigenes that resulted from herbivory by a xylem-feeding leafhopper, *Homalodisca coagulata*. Insect feeding led to a significant expression change in 50 transcripts broadly functioning in direct defense, defense signalling, ROS scavenging, transport, cell wall modification, photosynthesis, and abiotic stress. The authors also noted that the transcript profile recorded greatly resembled that induced by wounding, likely through JA-independent pathways. In contrast to similar studies with aphids, SA-dependent pathogenesis related genes were weakly induced.

Although transcriptional profiling using microarrays has developed into the most prominent tool for functional genomics, none has yet reported on the effects and responses of citrus to the major environmental constraints (salinity, flooding, water deficit, chilling, and iron deficiency). High-throughput analyses of gene expression in citrus challenged with major abiotic stresses, however, are currently underway in several laboratories around the world and will soon produce valuable information that might eventually lead to discovery of novel genes and functions. For instance, it is known that in citrus, physiological disturbances produced by salinity are associated with leaf chloride build up rather than with sodium accumulation, as observed in many plants [96, 97]. Genes in principle associated with the response of citrus to salinity were initially obtained from a cDNA expression library of citrus salt-treated cell suspensions. These genes, homologues to phospholipid hydroperoxides, glutathione peroxidases [98], olesins, Lea5, or lypoxygenases, were involved in the oxidative response rather than in the specific response to salinity. Other genes involved in oxidative stress well known in citrus are glutathione S-transferases [99] and copper/zinc-superoxide dismutases. However, recent microarray analyses are providing much-needed insights into chloride tolerance mechanisms and short- and long-term adaptation of citrus to salinity. Several teams are engaged in a Euro-Mediterranean MPC INCO project, started in 2006, between Spain, Morocco, Tunisia, Turkey, and France focused on tolerances to salinity and iron deficiency associated with alkaline soils. One of the major components of this project is the large-scale study and genome-wide acquisition of quantitative biological information on gene expression from multiple tolerant and susceptible genotypes. Work on transcriptomic comparisons in this area is confirming that Cl^−^ is the most important ion involved in the genetic response of citrus to salinity. In addition, major metabolic regulation changes are also apparent during salinity acclimatization in tolerant rootstocks. In contrast, flooding is mostly characterized by the rise of oxidative stress.

Chilling resistance in citrus is another area that has received much attention but lacks current comprehensive gene expression analyses provided by microarrays. Since most commercially important citrus varieties are cold-sensitive and therefore susceptible to freezing, *Poncirus trifoliata* (L.), an interfertile *Citrus* relative that can tolerate temperatures as low as −26°C after acclimation, is being used for improving cold tolerance in citrus rootstocks and as a source for the identification of cold-regulated genes. In general, many studies have been performed through subtractive hybridization [100] and DDRT-PCR [101] comparing expression in sensitive and resistant varieties. It has been shown, for example, that expression of a *C-repeat-binding factor* (*CBF*) and one of its targets, *COR19*, a cold-induced gene, accumulated both earlier and to higher levels in *Poncirus*. Moreover, COR19, COR11 [102], and COR15 were found to belong to an unusual group 2 LEA gene family responsive to low temperature. These dehydrins differ from most other plant dehydrins in having an unusual K-segment similar to that of gymnosperms and in having a serine cluster (S-segment) at an unusual position at the carboxy-terminus [103]. Citrus, however, also possesses the typical plant angiosperm-type K-segment consensus sequence. Other up-regulated transcripts that may play a role in cold sensitivity are a novel RING-H2 finger gene, AP2 domain containing genes and CTL, and a homologue of a low-temperature-responsive gene from *Arabidopsis*. During postharvest storage, chilling injury in citrus fruit can be reduced by previous short heat treatments that activate different molecular responses. Genes differentially expressed in the chilling response have mostly been related to lipid membrane and cell wall enzymes, to main regulators of secondary metabolism and hormonal homeostasis, and to oxidative and general stress responses [104, 105]. 

Although the main applications of microarrays to date are in transcriptome profiling analyses, microarrays can also be used to study DNA variation. Oligonucleotide arrays are particularly suited for the detection of single nucleotide mismatches during hybridization and, hence, for the discovery of novel DNA variants or the determination of known variants. The citrus GeneChip, for example, was designed to genotype 3219 genes using 5023 SNPs. The 20 K Spanish Consortium microarrays have been used to identify heterozygous deletions in fast neutron irradiated citrus mutants through array-based comparative genomic hybridization (array-CGH) and to study gene colinearity. Preliminary CGH yielded several candidate genes that were in haploid gene dosage. After comparison with the *Arabidopsis* and *Populus* genomes, it was observed that *Populus* orthologues of *Citrus* deleted genes grouped in two duplicated chromosomes in contrast to *Arabidopsis* orthologues that were distributed in several chromosomes (Ríos and Talon, unpublished data).

### 5.4. Genetic transformation

Citrus transformation procedures, in general, follow *Agrobacterium tumefaciens* protocols, and subsequent regeneration through organogenesis and somatic embryogenesis are also rather typical and straightforward [106]. Transformation efficiency of young material is usually low, 15–20%, and a major achievement to overcome the juvenility limitation was the direct transformation of adult material [107].

In citrus, genetic transformation is mostly being explored as an alternative to classical genetic breeding and not many examples can be found in the literature illustrating the use of genetic transformation for functional genomics. For example, there is interest in modulating the growth habit of rootstocks since this might eventually affect the development of the scion and facilitate diverse cultural practices (e.g., pruning, pesticide applications, and harvesting). Thus, it was known that the ectopic overexpression in tobacco of a citrus GA 20-oxidase, a regulatory step of gibberellin biosynthesis in citrus, [108] enhanced gibberellin content and shoot growth [109]. Later, Fagoaga et al. [110] generated transgenic Carrizo rootstocks overexpressing this GA 20-oxidase and confirmed that the gene controls gibberellin flux through the pathway since taller (sense) and shorter (antisense) phenotypes correlating with higher and lower levels of active GA_1_ were obtained. In these transgenic lines, however, cell division was more affected than cell elongation, in contrast to the effects observed in herbaceous plants [111]. In another example, an antisense construction with a citrus ACC synthase gene repressed ACC increase after a chilling treatment. A pectin methylesterase gene (*Cs-PME4*) isolated from sweet orange to prevent juice cloud separation was also introduced via protoplasts and subsequent regeneration through somatic embryogenesis [112].

Generally, characteristics related to commercial valuable traits are modified through the use of transgenes. To accelerate flowering time, Carrizo seedlings constitutively overexpressing the *Arabidopsis* floral-regulatory genes *LEAFY* (*LFY*) or *APETALA1* (*AP1*) were generated [113]. Both kind of transgenic citrus produced fertile flowers in their first year considerably shortening the juvenile phase. Consistently with the role of *LFY* and *AP1,* juvenility in citrus was positively correlated with CsTFL (homolog to TERMINAL FLOWER) transcript accumulation and negatively correlated with LEAFY and APETALA1 RNA levels [114]. In a similar approach but with a citrus gene, it was showed that transgenic *Poncirus* carrying the *CiFT* gene (homolog to *FLOWERING LOCUS T*), another flowering time gene, also exhibited early flowering although this phenotype was accompanied with several pleiotropic effects [115]. It is possible that the early flowering *AP1* and *CiFT* transgenic citrus could be used as rapid cycling genotypes for functional genomics studies. In a further example, Carrizo rootstock constitutively expressing a Δ^1^-pyrroline-5-carboxylate synthetase mutant gene from *Vigna*, showed higher water deficit tolerance [116]. Regarding tolerance to stresses, however, a huge amount of work has been centered on resistance to biotic stresses, a matter of major relevance in citrus industry. Thus, tolerance or resistance to *Phytophthora citrophthora*, the most widely spread oomycete in citrus growing areas, was generated by introducing the gene P23, that codes for a pathogenesis-related protein induced in tomato. These results provided evidence for the antifungal activity in vivo of the P23 pathogenesis-related protein against *P. citrophthora* [117].

A great effort is also being developed to understand the basis of the tolerance to citrus tristeza virus (CTV), the causal agent of the most important virus disease in citrus. The strategy is generally supported by the concept of pathogen-derived resistance (PDR), based on expression of viral sequences interfering with the virus life cycle in plants. CTV resistant transformants have been obtained by genetically engineering the *p25* and *p23* genes from CTV [118]. However, it still remains to be elucidated if transgenic citrus plants expressing CTV-derived sequences are a plausible alternative to cross protection to control CTV strains in the field. In an alternative strategy, heterologous expression of plant-derived resistance genes is promoted to confer resistance against CTV. General resistance to CTV has been found in *Poncirus trifoliata*, and a region containing the resistance gene (*Ctv*) has been characterized. Furthermore, work is under way for other pivotal diseases such as citrus mosaic virus (CiMV), citrus canker, and citrus blight.

### 5.5. Reverse genetics

In addition to genetic transformation, the capability to perform reverse genetic analyses is crucial to develop functional studies. The creation of transgenic lines is a powerful and straightforward way to determine gene function. However, in citrus, high-throughput transgenic programs such as the generation of RNA interference knockouts, activation tagging through enhancer elements, gene-trap T-DNA insertions, or transposable tagging systems have not yet been developed. The capacity for the maintenance and characterization of many transgenic lines of a perennial tree with both a long juvenile phase, large individual plant size, and a complex reproductive biology has probably hindered these developments. In *Populus*, however, activation tagging and insertional mutagenesis approaches are been explored despite logistical challenges in working with transgenic trees, a direction that may well be followed by citrus researchers in the near future.

### 5.6. Tilling/fast neutrons

Since gene disruption is the most effective method to analyze gene functions and no efficient tagging or insertional methods are available in citrus, strategies based on genome-wide mutagenesis such as TILLING (targeted induced local lesions in genomes) and fast neutron mutagenesis are being explored further [84]. These approaches are nontransgenic and have particular interest for the industry where the debate on GMOs has restricted their application in crop improvement. TILLING identifies individuals carrying point mutations while the fast neutron mutagenized population is searched for gene deletions using PCR amplification. Both approaches, at the moment, are of limited usefulness as strategies for reverse genetics in citrus because of the lack of genomic sequences and the large amounts of space required for mutated populations of suitable size. ECOTILLING, however, on natural citrus variants and microarray-based detection of deletions on fast neutron citrus mutants in a more direct genetics strategy are very straightforward approaches. However, unless a high-throughput transformation protocol is developed for citrus, functionally analyzing all genes with tagging approaches or genome-wide mutagenesis and screening are not realistic strategies.

### 5.7. Viral-induced gene silencing

Viral-induced gene silencing (VIGS), on the other hand, is an attractive and very promising alternative in citrus. Knocking out the expression of a gene by VIGS does not require genetic transformation and has proven to be a very efficient tool for function analysis of plant genes. VIGS is particularly suitable for woody plants like citrus with long juvenile periods that require long periods between transformation and fruiting. In a hopeful work, Dr. Guerri and colleagues at IVIA have recently showed that VIGS might be possible in citrus using *Citrus leaf blotch* *virus* (CLBV) as a viral vector (Dr. Guerri, personal communication). These workers cloned a full-length cDNA of the CLBV genome [119] in a binary vector under the control of the 35S promoter and demonstrated that tobacco and citrus plants Agro-infiltrated with this construct became infected and replicated CLBV normally. Recently, they have showed that tobacco plants Agro-infiltrated with a CLBV chimeric construct carrying a fragment of the phytoene desaturase gene developed photobleaching symptoms and reduced the cognate transcripts. Parallel experiments in citrus are planned. Availability of the CLBV-based vector will certainly open new possibilities to study functional genomics in citrus.

### 5.8. Proteomics/metabolomics

Other powerful approaches for functional genomics studies such as proteomics and metabolomics to comprehensively analyze proteomes and phenotypes have just begun for citrus. For example, Blumwald and coworkers (UCD) are using two main approaches, namely 2D gel analyses coupled with MALDI-TOF-TOF from juice sac cell vacuoles and LC^2^-MS-MS analyses of ER/Golgi, plasma membrane, tonoplast, mitochondria, and soluble enriched fractions from citrus juice sac cells to define the “citrus fruit proteome.” Current work is in progress but they have already reported the identification of over 1500 proteins involved in sugar metabolism, citrate cycle, signalling, transport, and processing and have characterized changes in protein expression during development [120]. In a further example, proteome changes in the fruit albedo during postharvest ageing were studied through 2D-PAGE, and relevant proteins were also identified through mass spectrometry determinations [121]. This proteomic survey indicated that major changes in protein content (ATP synthase beta subunit, ascorbate peroxidase, translationally controlled tumor protein, cysteine protease, etc.) were apparently related to the activation of programmed cell death.

Numerous analyses of citrus metabolites, especially of ripening and matured fruits, have been reported in the past. However, new methods to characterize the metabolic phenotypes of representative lines from mutants and natural varieties must be developed. Metabolic profiling and metabolomic procedures using state-of-the-art gas chromatography-mass spectrometry or fast gas chromatography-time-of-flight mass spectrometry need to be setup.

The final objective of citrus functional genomics is to identify candidate genes, alleles, and genotypes improving citrus fruit quality, correlating phenotypic analyses, metabolomic profiling, and gene expression. At completion, genes and alleles with major functions in nutritional quality could be selected and genotypes with improved fruit composition searched among existing collections or generated.

## 6. CONCLUSION AND FUTURE PROSPECTS

This paper has reviewed various aspects of the current status of citrus genome research, including the development of fundamental tools, the applications currently under way and envisaged leading to solutions to seemingly intractable problems facing the citrus industries of the world, the opportunities of improving further the perceived and real value of citrus fruit and products, and the challenges that remain not only for genomic research but for making progress in truly incorporating new knowledge into new plant materials. The international citrus research community has been growing closer together, and new international alliances are making the achievement of truly great advances possible; this is essential, as no one group or even nation has sufficient resources to address all the needs for tool development and deployment, and many of the problems faced are global in nature. It is clearly evident that by combining research resources and by adopting the principle of depositing information in the public domain, freely available to global research partners, the promise of genome research to improve citrus plants, production, and protection from diseases, and enhanced product quality and value, can be realized. The free availability of these tools and materials is truly the key to the success in genomics research. Citrus is a very important tree fruit crop throughout the world not only is it of great economic significance but it is also of great value for human nutrition and well-being. In addition, it possesses many unique characteristics of great biological interest. Consequently, the benefits of an expanded and focused effort into all aspects of citrus genomics will be of great benefit to humanity in general as well as to the realm of plant science. Citrus will have a first genome sequenced in the very near future; this will not be the end of the process but the beginning of many more citrus genome sequencing projects to add layers of valuable information to the already developed and developing tools to understand the functions and interrelationships of genes, their products, and their interactions with the environment. Through the acquisition of this knowledge and its application to the field, citrus will continue to be an economically valuable fruit crop plant and a source of important health and nutrition benefits to people throughout the world.
